# Impact of COVID-19 Pandemic on STEMI Networks in Central Romania

**DOI:** 10.3390/life11101004

**Published:** 2021-09-24

**Authors:** Roxana Hodas, Imre Benedek, Nora Rat, Istvan Kovacs, Monica Chitu, Theodora Benedek

**Affiliations:** 1 Pharmacy, Science and Technology of Targu Mures, George Emil Palade University of Medicine, 540142 Tirgu Mures, Romania; roxana.hodas@umfst.ro (R.H.); imre.benedek@umfst.ro (I.B.); istvan.kovacs@umfst.ro (I.K.); monica.chitu@umfst.ro (M.C.); theodora.benedek@umfst.ro (T.B.); 2 Clinic of Cardiology, Emergency Clinical County Hospital, 540136 Tirgu Mures, Romania

**Keywords:** COVID-19, STEMI network, acute myocardial infarction

## Abstract

The COVID-19 pandemic has had a major impact on cardiovascular emergencies. The aim of this study was to investigate the impact of the COVID-19 pandemic on a regional network for management of ST-segment elevation acute myocardial infarction (STEMI). Methods: We report a single center’s experience of patients hospitalized for ACS in a high-volume hub of a STEMI network during the lockdown (in the first pandemic trimester), compared with the same time interval of the previous year and including all consecutive patients referred for an AMI during the second trimester of 2020 (from April to June) or during the same time interval of the previous year, 2019. Results: The absolute number of hospital admissions for AMI decreased by 22.3%, while the non-AMI hospitalizations decreased by 77.14% in Q2-2020 compared to Q2-2019 (210 vs. 48, *p* < 0.0001). As a consequence, the percentage of AMI cases from the total number of hospital admission increased from 38% to 68% (*p* < 0.0001), AMI becoming the dominant pathology. In the STEMI group there was a significant reduction of 55% in the absolute number of late STEMI presentations. Functionality of the STEMI network at the hub level did not present a significant alteration with only a minor increase in the door-to-balloon time, from 34 min to 41 min. However, at the level of the network we recorded a lower number of critical cases transferred to the interventional center, with a dramatic reduction of 56.1% in the number of critical STEMI cases arriving in the acute cardiac care unit (17.0% vs. 7.3%, *p*-0.04 for KILLIP class III, and 21.17% vs. 11.11%, *p* = 0.08 for resuscitated out of hospital cardiac arrest). Conclusions: The COVID-19 outbreak did not have a major impact on the interventional center’s functionality, but it limited the capacity of the regional STEMI network to bring the critical patient with complicated STEMI to the cathlab in time during the first months of the lockdown. Even a very well-functioning STEMI network like the one in Central Romania had difficulties bringing the most critical STEMI cases to the cathlab in time.

## 1. Introduction

The COVID-19 pandemic outbreak challenged healthcare systems worldwide in an unprecedented manner [1]. As a result, treating patients with critical non-COVID conditions became problematic in many healthcare systems. Lockdowns have been effectively implemented to limit the spread of SARS-CoV-2 in numerous countries [2,3]. Whilst lockdowns slowed the progression of the pandemic, they can also involve undesired consequences in treatment of non-COVID emergencies, such as acute myocardial infarction (AMI).

In patients suffering an AMI, survival depends on the timely initiation of reperfusion therapy. Especially in the case of ST-elevation myocardial infarction (STEMI), current guidelines recommend primary percutaneous intervention (pPCI) in the first 2 h after presentation to the hospital, which means that the STEMI patient should arrive in the closest cathlab within the recommended timeframe of 2 h. Therefore, STEMI networks have been implemented in the attempt to get the STEMI patient to the cathlab as soon as possible. A typical STEMI network has an interventional center with a cathlab serving as a hub for affiliated spoke centers. Implementation of STEMI networks has been proven to dramatically reduce STEMI-related mortality, since they manage to bring the patient to the cathlab in time to receive life-saving pPCI [4].

To be effective, the hub of a STEMI network should have not only an experienced interventional team in the cathlab, but also a professional acute cardiac care unit (ACCU) able to provide acute monitoring and treatment in the immediate post-revascularization period. This kind of ACCU deals with acute coronary syndromes (ACS) in approximately 52% of admitted cases, while the other 48% of the pathology treated in the ACC is represented by acute heart failure, arrhythmia, pulmonary embolism, etc [5]. Development of ACCUs linked to interventional centers in regional STEMI networks are proven to have a significant contribution to reduction of STEMI mortality [6].

Early reports presented a direct influence of COVID-19 on ACSs, as previous infections with respiratory viruses may lead to a higher risk of ACS throughout the pro-thrombotic infectious status [7]. At the same time, COVID-19 increases the risk of ACS due to the cardiovascular inferences of virus-related systemic inflammatory status [8,9].

However, real-time reports indicated that severe actions taken by governments to contain the contagion led to a drop in the number of patients presenting to hospitals for emergency conditions, including ACS [10,11,12,13]. There was a dramatic decrease in ACS, by up to 40% in many regions around the world, especially in industrialized countries [14,15,16]. In parallel, an increased incidence of out-of-hospital cardiac arrests (OHCA) was reported, and a notable delay of emergency activations STEMI cases [17,18,19].

The aim of the study was to investigate the impact of the COVID-19 pandemic on a regional STEMI network. Specifically, we aimed to study: (1) how the new coronavirus affected the rate of AMI presentations to emergency care, (2) the impact of lockdown on the core activity of the ACCU serving as hub of a STEMI regional network, and (3) the functionality of a well-organized STEMI network during the lockdown.

## 2. Materials and Methods

In this study, we report a single center’s experience of patients hospitalized for ACS in a high-volume hub of a STEMI network during the lockdown (in the first pandemic trimester), compared with the same time interval of the previous year.

The study was conducted in the Clinic of Cardiology of the County Clinical Emergency Hospital Tirgu Mures, University of Medicine and Pharmacy Tirgu Mures, a clinic that represents the core of the first regional STEMI network created in Romania in 2005, covering a territory of 250 km and a population of approximately 1 million people in Central Romania. The workflow of this clinic during the last 5 years, 2015–2019, in terms of acute myocardial infarction, consists of an average value of 475 primary PCIs for STEMI and 620 PCI for NSTEMI.

### 2.1. Data Collection

In this study, we included all consecutive patients referred for an AMI in the ACCU of the County Clinical Emergency Hospital Tirgu Mures during the second trimester of 2020 (from April to June) or during the same time interval in the previous year, 2019. AMI was defined using the fourth universal definition of acute myocardial infarction and cross-checked with the International Classification of Disease 10th revision (ICD-10) codes. AMI admissions were classified as STEMI or non-STEMI according to the recorded ICD-10 codes.

### 2.2. Data Analysis—Study Endpoints

We compared all data related to admission, diagnosis, demographics, therapeutic management, and in-hospital outcomes in Q2-2020 (April, May and June 2020) to the same three months of the non-pandemic year, Q2-2019.

Specifically, we investigated:

(1) The impact of the COVID-19 pandemic on the absolute number of presentations for AMI in our regional system of care;
(2) The way in which COVID-19 pandemic changed the core activity of ACUU;
(3) The impact of the COVID-19 pandemic on the functionality of the regional STEMI network.

### 2.3. Ethical Aspects

The study protocol was performed in accordance with the Declaration of Helsinki statements as requested by the local hospital Ethical Committee, Study approval no. 11562/27.04.2021; all data were collected anonymously; all patients routinely subscribe to a disclosure statement for the use of personal data at the beginning of hospitalization.

### 2.4. Statistical Analysis

All analyses were conducted using GraphPad Prism version 9.0 Software (GraphPad Software, San Diego, CA, USA).

Differences among the two study periods were assessed using the χ2 test.

Descriptive statistics for categorical variables were reported by frequency and percentage, and Pearson’s chi-square was used. Continuous variables were presented as mean ± standard deviations. Comparison of continuous data was tested using Student’s *t*-test or Mann–Whitney U test where appropriate.

All *p*-values were two tailed and a *p*-value of <0.05 was considered significant.

## 3. Results

### 3.1. Decrease of Emergency Presentations for Acute Cardiac Conditions during COVID-19 Lockdown

In total, 455 patients were admitted to the Clinic of Cardiology for different types of cardiovascular emergencies in Q2-2019 or Q2-2020. From these 455 cases, 231 patients with confirmed AMI (STEMI or non-STEMI type) were included in this analysis.

The main results of the comparison between the pre-pandemic and pandemic years are presented in Table 1.

**Figure 1:**
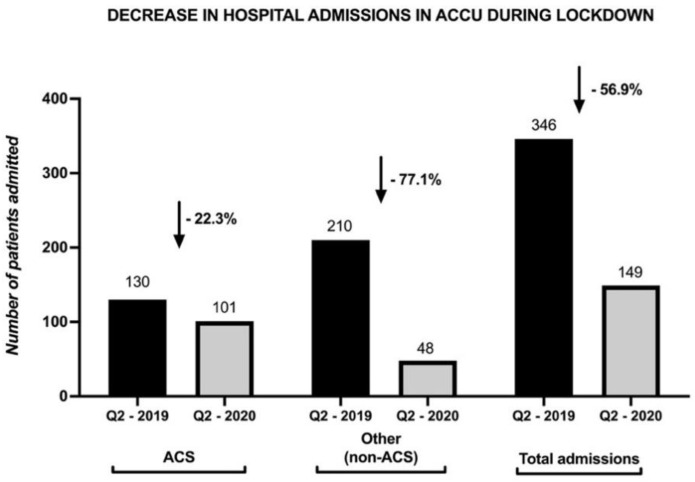
Decline in all kinds of hospital admissions, especially in non-ACS admissions, during pandemic trimester (Q2-2020) compared to non-pandemic trimester (Q2-2019). ACS, acute coronary syndrome; non-ACS, non-acute coronary syndrome. The arrow shows the decreasing trend between the compared trimesters.

The absolute number of hospital admissions for AMI decreased from 130 in Q2-2019 to 101 in Q2-2020, demonstrating an absolute reduction of 22.3% in the rate of presentation for AMI. The non-AMI hospitalizations drastically decreased by 77.14% (from 210 in Q2-2019 to 48 in Q2-2020, *p* < 0.0001) (Figure 1). As a consequence, the percentage of AMI cases from the total number of hospital admission increased from 38% in the pre-pandemic year to 68% in the pandemic period (*p* < 0.0001), AMI becoming the dominant pathology in the cardiology clinics.

However, there was no change in frequency of STEMI as a proportion of total AMIs during the pandemic trimester of 2020 (62.37%) as compared with the second trimester of 2019 (65.38%) (*p* = 0.7).

**Figure 2:**
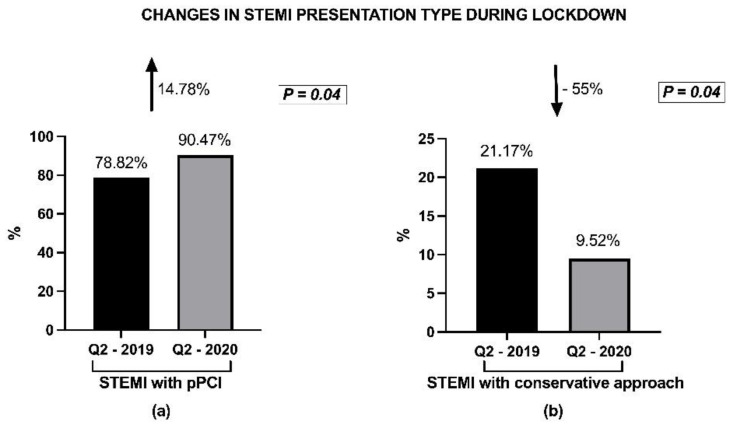
Change in STEMI presentation type during lockdown (Q2-2020) compared to the previous period (Q1-2019). (**a**) Increase in the percentage of patients presenting <12 h from symptoms onset, reported to the total number of STEMI presentations; (**b**) Significant decrease of the percentage of STEMI late presentation (patient presented >12 h from symptoms onset) during pandemic months. The arrow shows the increasing and decreasing trend between the compared trimesters.

Interestingly, in the STEMI group there was a 14.7% increase in the proportion of patients who presented to hospital in time for pPCI (within the recommended time interval of 12 h after the onset of symptoms) during the lockdown period. Therefore, pPCI was possible in 90.47% of STEMI cases during Q2 of 2020, as compared to only 78.82% reported during Q2 of 2019 (*p* = 0.04) (Figure 2). However, this was not related to an absolute increase in the number of timely presentations for STEMI, but rather to a significant reduction of 55% in the absolute number of late STEMI presentations (after the first 12 h after the onset of symptoms) in Q2-2020, since during lockdown patients who stayed home more than 12 h were more likely to continue to stay home instead of going to ER, from the fear of the virus.

### 3.2. Patient Characteristics

There were no significant differences in AMI patient profile recorded for STEMI population during the pandemic Q2 compared to Q2-2019. The mean age was 63.95 in Q2-2020 vs. 64.93 in Q2-2019 (*p* = 0.72) in STEMI patients, and 65.22 in Q2-2020 vs. 66.26 in Q2-2029 (*p* = 0.74) in non-STEMI population. The proportion of male patients was similar in Q2-2020 and Q2-2019 among STEMI cases (63.23% vs. 56.38%, *p* = 0.38), as well as among total hospital admissions (60.60% in Q2-2020 vs. 61.58% in Q2-2020, *p* = 0.88).

However, within the non-STEMI subgroup, a significantly lower proportion of males was registered in Q2 of 2020 (37.14%) compared with Q2 of 2019 (69.64%) (*p* < 0.0001). At the same time, the highest reduction of emergency admissions was recorded among male patients with non-STEMI (46.66% reduction, *p* < 0.0001), much higher than the reduction in STEMI hospitalizations or in total hospital admissions.

### 3.3. Change of the Core Activity of the Regional Acute Cardiac Care Unit

A significant shift of the core activity of the ACCU was observed during the pandemic months, towards a more specific ‘AMI profile’ of the patients. The proportion of AMI cases from total hospital admissions substantially increased from 37.5% in Q2-2019 to 67.78% during the pandemic Q2 (*p* < 0.0001), showing an obvious shift in the core activity of the ACCU, which began to look more like a “AMI unit”.

### 3.4. Functionality of the Regional STEMI Network during Lockdown

Functionality of the STEMI network at the hub level did not show significant alteration as a result of the COVID-19 pandemic and associated lockdown. Several changes were implemented in the triage protocols and patient circuits, in order to protect the personnel. All patients were screened for COVID infection prior to admission in the cathlab, and all patients were treated as potentially positive until a negative RT-PCR test was received. Specific protocols for the triage of ACS patients were implemented into the network at different levels, in order to strictly separate COVID-19 and non-COVID-19 patients into separate management pathways and environments, and all medical personnel wore personal protective equipment.

In our center, door-to-balloon times were similar between years, highlighting that COVID-19 protective measures had no impact on STEMI management and therefore do not explain the increase in total ischemic times. A minor increase in the door-to-balloon time from 34 min to 41 min was recorded, as a result of the increased time spent on personal protective measures in parallel with the implementation of specific protocols to separate COVID-19 from non-COVID-19 cases.

**Figure 3:**
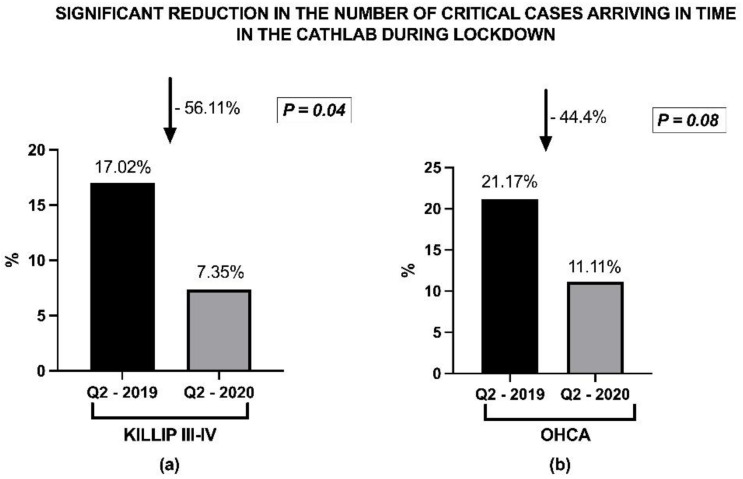
Impact of lockdown on the ability of the STEMI network to bring complex cases to interventional center. (**a**) STEMI with KILIP class III-IV. A significant decrease in STEMI cases presenting in KILLIP class III-IV who arrived to the cathlab was encountered during the COVID-19 pandemic trimester compared to the control trimester of 2019; (**b**) STEMI complicated with cardiac arrest. A significant reduction in the number of STEMI cases complicated with cardiac arrest who arrived to the cathlab was recorded in the pandemic trimester compared with the same period in 2019. The arrow shows the decreasing trend between the compared trimesters.

However, at the level of the network we recorded a lower number of critical cases transferred to the interventional center, with a dramatic reduction of 56.1% in the number of critical STEMI cases arriving in the acute cardiac care unit in Q2-2020 compared to Q2-2019. The proportion of STEMI cases with KILLIP class III or IV decreased from 17.0% in Q2-2019 to 7.3% in Q2-2020 (*p* = 0.04), while hospital admission for cardiac arrest complicating STEMI decreased from 21.17% in Q2-2019 to 11.11% in Q2-2020 (a 44.4% reduction, *p* = 0.08) (Figure 3).

As a result of the lower number of late STEMI presentations (at more than 12 h after the onset of symptoms), and the lower number of critical cases arriving in the interventional center, in-hospital STEMI mortality in the interventional center decreased from 4.9% in Q2-2019 to 3.8% in Q2-2020 (21.7% relative reduction), showing an artificial reduction of STEMI-related in-hospital mortality during lockdown. This was in reality caused by the dysfunctionality of the STEMI network, which in some cases failed to bring complex cases to the interventional center.

## 4. Discussion

### 4.1. Hospital Admissions for ACS during COVID-19 Pandemic

Since the beginning of the COVID-19 pandemic, an unexplained reduction in ACS presentations, by 25–48%, was reported both in Europe [11,17,18,19] and the United States [14,20], despite the fact that patients still suffered from STEMI in this period. These results were generally constant across six continents and, though grounded by self-reported observations, they are reinforced by objective evidence from European and US registries suggesting a 25–40% average decrease in STEMI activations through the COVID-19 outbreak [11,14,19,20,21,22]. Overall, these records describe a picture of nearly half of patients with ACS not reaching the EMS and not receiving timely treatment. This was also observed in our study, with a reduction of 22.3% in the rate of presentation for AMI. However, it is interesting to note that the most dramatic impact of the lockdown was recorded in non-AMI patients, with a reduction of 77.14% of non-AMI presentations to the emergency department in this study.

As previous described, the main reason for the decrease in STEMI activations consists in the fear of infection and limited medical information concerning this pathology. In such circumstances, telehealth could represent a viable option for contact between patients and physicians in acute conditions, and previous observational studied described that telemedicine can reduce pre-hospital delays for pPCI. However, telemedicine is not really implemented yet in Romania, therefore this medical tool could not have been used in this pandemic situation.

This made AMI a very dominant pathology in our ACCU, substantially changing the core activity of our clinic in to a more “AMI profile” as a consequence of the pandemic. It is important to note that during the pandemic outbreak, official regulations asked the emergency service to redistribute non-AMI cases to non-PCI centers in order to reduce the risk of cathlab contamination, thus protecting the functionality of pPCI centers. Since in Romania for a long period of time RT-PCR results were not available in 24 h, patients with uncertain infective status but with an acute cardiac condition not requiring PCI were redistributed to hospitals with acute cardiac care facilities but without a cathlab, while pPCI centers received only the major emergencies requiring interventional therapy. This helped pPCI centers to reduce the risk of contamination, limiting their activity only to AMI patients and led to the AMI profile of ACCU patients in pPCI centers.

### 4.2. STEMI Networks, Critical Network Times and Lockdown

Functionality of the STEMI networks is crucial for appropriate management of cardiovascular emergencies. In acute cardiac care, the time from symptom onset to treatment is one of the most powerful predictors of survival, and STEMI networks are the only effective way to bring the patient in time for life-saving treatment. Implementation of the regional STEMI network in Central Romania in 2004 resulted in a significant increase in the rate of pPCI, from 10.88% in 2004 to 78.64% in 2011, which was directly associated with a major decrease in the in-hospital mortality rate of STEMI patients, from 20.73% in 2004 to 6.35% in 2011 [4]. Functionality of this STEMI network in Central Romania under pandemic conditions was analyzed in this study, which identified a well-functioning system at the hub level, but with difficulties in bringing complicated cases from the territorial centers to the cathlab during lockdown. In other research, Chew et al. also addressed the issue of pPCI during pandemic and reported relevant delays in door-to-balloon times during the COVID-19 outbreak [23]. In addition to this research, our article evaluates the functionality of each structural component of the STEMI network during the COVID-19 pandemic, not only the efficiency of the PCI center. Specifically, our study shows how the pandemic outbreak severely altered the capacity of a STEMI network at regional level to bring complicated cases to the interventional center.

Several recent studies reported large delays from diagnosis to pPCI during COVID-19 waves, with a three-fold increase in the ischemic time. This was mainly attributed to a concerning larger period spent from the onset of symptoms to FMC, a period which is patient-dependent [7,24]. The average time from symptom onset to FMC among patients with STEMI was reported as almost two-fold longer during the COVID-19 outbreak as compared to the control period (112 min vs. 60 min, *p* < 0.05) [25]. A study on the management of infarctions in China during the COVID-19 pandemic showed that the time elapsed from symptom onset to FMC increased from an average of 82 min before the pandemic to an average of 318 min during pandemic [11]. The main proposed reason was related to patients’ hesitancy to go to the emergency room or to activate the emergency medical system, on the basis of the worldwide statement “do not come to the hospital” [7]. This fact introduced a novel “Covid-19-related delay” in the so-called “total ischemia time”.

Interestingly, in our study the proportion of patients who presented to the hospital within the first 12 h after symptoms onset being eligible for pPCI increased by 14.7% during lockdown. This observation needs careful analysis, since it may appear to contradict other studies. However, data analysis reveals that there was no increase in the number of timely presentations for STEMI. It was only the percentage of timely presentations out of the total number of STEMI which increased significantly, in parallel with a dramatic reduction of 55% in the absolute number of late STEMI presentations (after the first 12 h after the onset of symptoms) during lockdown period. This may be explained by the fact that patients with less severe symptoms avoided contact with the emergency system, and those who resisted and stayed home for more than 12 h were more likely to continue to stay home instead of going to the hospital after more than 12 h, from the fear of the virus. In line with this observation are the main findings of a Swiss group’s research on the impact of COVID-19 on ACS, who reported that approximately one third of patients delayed their call to the emergency services due to fear of contracting COVID-19 in the event of hospital admission [25]. As in the first pandemic trimester, analyzed in the study, no official regulation was made by the health authorities to initiate thrombolysis and we did not record any increase in the number of thrombolysis performed.

It seems that changes in the perception of urgent care during the COVID-19 pandemic increased the delay between symptom onset and first medical contact in patients with STEMI [25].

### 4.3. Non-STEMI Management during Pandemic

The overall incidence rate of ACS undergoing PCI during the COVID-19 period was significantly lower in our study, driven by a three-fold lower incidence among NSTEMI-ACS patients in comparison with the control period [25]. This is in line with other studies that found significant reductions in non-STEMI presentations during lockdown. Chan et al. reported a reduction in the number of activations for confirmed ACS especially due to a decrease in NSTEMI and unstable angina (UA) cases during the COVID-19 lockdown period in New Zealand, while no differences were reported for STEMI hospital admissions [26]. These results may suggest the fact that during the lockdown period patients with less severe symptoms may have avoided presenting to emergency departments [25].

### 4.4. Gender Issues in ACSs during COVID 19 Pandemic

The gender issue in the COVID-STEMI patients have not been elucidated so far. Results from some previous studies found no difference between genders in the reduction of AMI presentations during the COVID-19 pandemic [10,27,28], whereas other studies suggested larger reductions in incidence among men or larger reductions among women [29,30].

In our study, male patients had a significantly lower proportion in Q2 of 2020 (37.14%) compared with Q2 of 2019 (69.64%) (*p* < 0.0001), and male patients with non-STEMI presented a 46.66% reduction of emergency admissions, the highest reduction recorded. This indicates that the male gender of this region has a higher risk of non-presentation during pandemic waves. Interestingly, the Romanian RO-STEMI registry indicated that women with STEMI presented later and benefit to a lesser extent from optimal interventional treatment. Female gender was an independent negative predictor of time from symptoms onset to thrombolysis within 2 h, and PPCI was about twice as frequent among male patients as among women [31]. In our study male patients are more sensitive to fear from the virus. Taken together, this shows that males in Romania might behave differently from females in terms of health-seeking behavior.

### 4.5. Network Functionality for Complex STEMIs during Lockdown

While the admission rates of true ACS cases have fallen since the pandemic outbreak, STEMI fatality rate and complications registered a substantial increase during the pandemic period compared to 2019 [32].

Reported patient-dependent delays in total ischemic time showed rose to a higher rate of major adverse cardiac events during the pandemic period [33]. In Italy, most of the multicenter studies returned an alarming image of almost half of AMI patients not reaching out to the hospital at all [32], complemented by a threefold increase in mortality and complications, even though ACS management protocols were promptly implemented [34].

At the same time, a remarkable increase in OHCA rate was observed, particularly in states with a higher incidence of COVID-19 cases, with very alarming issues highlighted regarding the dramatic decline in survival of OHCA patients, both in countries most affected by the pandemic and in those affected only marginally [35,36]. The Lombardia Cardiac Arrest Registry reported a 58% increase in OHCA during the first 40 days of the COVID-19 outbreak [17,19]. Similarly, a study conducted by Nils et al. in Switzerland reported significantly more patients presenting with OHCA (22.2% vs. 7.1%, *p* < 0.01) during the COVID-19 period compared with the control period [25].

The decrease in the number of patients in the KILLIP III-IV class observed in our study may be partly explained by reluctance in seeking medical assistance in the very early phase of ACS after the onset of symptoms, postponing hospital admission. However, since these critical cases are less exposed to a subjective interpretation of their symptoms, taking into consideration their critical condition, a more probable explanation is that for logistical reasons the network failed to bring a certain number of complex cases to the cathlab, or did this with a significant delay. This may be related to the delay in transportation (since the ambulance system was overwhelmed), the delay at the level of spoke units, waiting for the results of PCR tests before transferring the patient, or difficulties in arranging immediate transportation in cases with unconfirmed diagnosis and uncertain infection status.

As the time intervals at the interventional center presented only minor increases fully explainable by the need for additional personal protective measures, we can say that the COVID-19 outbreak did not have a major impact on the interventional center’s functionality, but rather limited the capacity of the regional STEMI network to bring the critical patient with STEMI and major complications to the cathlab in time during the first months of lockdown.

## 5. Conclusions

The COVID-19 pandemic had a strong impact on the functionality of the STEMI network in Central Romania but did not affect the functionality of the interventional center. Even a very well-functioning STEMI network like the one in Central Romania had difficulties bringing the most critical STEMI cases to the cathlab in time. More efforts are necessary to adapt the organization of the STEMI network to forthcoming challenges, in order to provide medical treatment in accordance with the current guidelines even under very complex conditions.

## Figures and Tables

**Table 1:** AMI patient characteristics, management, and in-hospital outcomes in the ACCU of the STEMI network during lockdown, compared to similar non-COVID period of the previous year.

	Period		
	Previous Year Control Q2-2019	Case Report Q2-2020	*p*-Value
*All admissions*			
Total admissions	346	149	
Non-ACS	210	48	
Male gender (%)	61.58	60.60	0.88
*Acute Coronary Syndromes Hospitalizations*			
ACS (STEMI + NSTEMI)	130	101	
% ACS from Total Admissions (%)	37.57	67.78	**<0.0001**
% STEMI from ACS	65.38	62.37	0.76
*STEMI treatment approach*			
% STEMI with PCI from total STEMI	78.82	90.47	**0.04**
% conservative STEMI from total STEMI	21.17	9.52	**0.04**
% conservative STEMI from total admissions	5.20	4.02	>0.99
*Cardiac Arrest*			
% CA from total admissions (%)	9.53	9.39	>0.99
% STEMI with CA from total STEMI (%)	21.17	11.11	0.08
% NSTEMI with CA from total NSTEMI (%)	2.22	13.15	**0.005**
*Mortality rate*			
STEMI (%)	15.29	6.34	0.06
STEMI with PCI (%)	4.91	3.84	>0.99
NSTEMI (%)	2.22	5.26	0.44
*STEMI patient’s demography*			
Male gender (%)	56.38	63.23	0.38
Mean age	64.93	63.95	0.72
*NSTEMI patient’s demography*			
Male gender (%)	69.64	37.14	**<0.0001**
Mean age	66.26	65.22	0.74

Significant values are written in bold. ACS = acute coronary syndrome; CA = cardiac arrest; STEMI = ST-segment elevation myocardial infarction; NSTEMI = non-ST-elevation myocardial infarction; PCI = percutaneous coronary intervention.
